# Supplementary material for: Economic Trends in Commonly Used Drugs for Spinal Fusion and Brain Tumor Resection: An Analysis of the Medicare Part D Database
Source: Biomedicines. 2023 Aug 3;11(8):2185. doi: 10.3390/biomedicines11082185 (PMC10452193; doi:10.3390/biomedicines11082185)
Supplement: Supplementary file 1 [file biomedicines-11-02185-s001.zip › Drug Data, Pearson's and Linear Regression/procedural data/Procedural Codes Quiered.pdf]

Spinal Procedures:

22551 – Fusion of spine bones with removal of disc at upper spinal column, anterior approach
22552 – Fusion of spine bones with removal of disc in upper spinal column below second vertebra of neck, anterior approach
22554 – Fusion of spine bones with removal of disc at upper spinal column, anterior approach
22558 – Fusion of spine bones with removal of disc at lower spinal column, anterior approach
22585 – Fusion of spine bones with removal of disc, anterior approach
22595 – Fusion of spine bones at skull base, posterior approach
22600 – Fusion of upper spine bones, posterior or posterolateral approach
22610 – Fusion of middle spine bones, posterior or posterolateral approach
22612 – Fusion of lower spine bones, posterior or posterolateral approach
22614 – Fusion of spine bones, posterior or posterolateral approach
22630 – Fusion of lower spine bones with removal of disc, posterior approach
22632 – Fusion of lower spine bones with removal of disc, posterior approach
22633 – Fusion of lower spine bones with removal of disc, posterior or posterolateral approach
22634 – Fusion of lower spine bones with removal of disc, posterior or posterolateral approach
22800 – Fusion of spine bones for correction of deformity, posterior approach, up to 6 vertebral segments
22802 – Fusion of spine bones for correction of deformity, posterior approach, 7 to 12 vertebral segments

Neurosurgical Procedures:

61510 – Removal of brain tumor
61512 – Removal of brain tumor
61520 – Removal of eighth cranial nerve brain tumor
61548 – Excision of pituitary gland tumor
61580 – Removal of nasal sinuses to approach brain lesion
61582 – Removal of facial bone to approach brain lesion
61584 – Removal of facial bone to approach brain lesion
61592 – Removal of cheek bone and skull to approach brain lesion
61608 – Removal of lesion at skull base
62165 – Removal of pituitary gland tumor using an endoscope
